# Viscosupplementation in Degenerative Joint Disease: Current Evidence, Indications, and Clinical Outcomes

**DOI:** 10.7759/cureus.106385

**Published:** 2026-04-03

**Authors:** Maximiliano Cueva Pérez, Antonio Cueva Pérez, Antonio Cueva Aldana

**Affiliations:** 1 Orthopaedics and Traumatology, Faculty of Medicine, Universidad Autónoma de Guadalajara, Guadalajara, MEX; 2 General Practice, Universidad Autónoma de Guadalajara, Guadalajara, MEX; 3 Orthopaedics and Traumatology, Universidad Autónoma de Guadalajara, Guadalajara, MEX

**Keywords:** hyaluronic acid, joint preservation, knee osteoarthritis, osteoarthritis management, treatment gap, viscosupplementation

## Abstract

Intra-articular hyaluronic acid (IA-HA), or viscosupplementation, remains a subject of ongoing debate in the non-surgical management of knee osteoarthritis. Despite conflicting clinical guidelines, its use continues to grow due to a favorable safety profile and its role in addressing the “treatment gap” for patients with Kellgren-Lawrence grades II and III. This structured narrative review identifies and synthesizes evidence from PubMed, Scopus, and recent clinical guidelines (2006-2026) to explore the physiological role of IA-HA, emphasizing its rheological properties and the dual mechanism of viscosupplementation and viscoinduction. We evaluate current clinical evidence, acknowledging the inherent heterogeneity of reported outcomes while discussing the potential for symptomatic relief that may, in selected populations, extend beyond the traditional six-month window. Furthermore, this work addresses the social and economic relevance of IA-HA, specifically its opioid-sparing benefits amidst the global opioid crisis and its potential to delay total knee arthroplasty (TKA). By refining patient selection and understanding the impact of molecular weight and combination therapies, clinicians can optimize outcomes. This review provides an evidence-based framework for integrating IA-HA into a multimodal joint preservation strategy to improve quality of life and reduce the global burden of degenerative joint disease.

## Introduction and background

Degenerative joint disease as a global health burden

Degenerative joint disease, predominantly manifested as knee osteoarthritis (OA), has evolved into one of the most critical public health challenges of the 21st century. It is currently the leading cause of chronic disability in the adult and elderly population worldwide, affecting more than 250 million individuals [1]. However, these global estimates should be interpreted with some caution, as substantial variation in diagnostic criteria (clinical vs. radiographic) and reporting standards across regions may lead to under- or overestimation of true disease prevalence [2]. Epidemiological data suggest a concerning trajectory; it is estimated that one in two individuals will develop symptomatic knee OA in at least one joint by the age of 85 [3]. This prevalence is expected to rise sharply due to the dual impact of an aging global population and the increasing incidence of obesity, which places a mechanical strain on weight-bearing joints [1,4,5].

Beyond the physical impairment, the economic burden of OA is massive. In developed nations, the total costs associated with managing OA (including medical treatment, surgical interventions, and long-term care) account for between 1% and 2.5% of their gross domestic product (GDP) [3,4], which is heavily driven by end-stage treatments. Nevertheless, cross-country comparisons of economic impact should be interpreted cautiously, as differences in healthcare financing models and cost-accounting methodologies can substantially influence reported expenditure [6]. For instance, the annual cost of total knee arthroplasties in the United States alone exceeds $27 billion. The rising incidence of TKA procedures poses a significant burden on healthcare systems globally, highlighting the need for strategies that can effectively manage chronic pain while potentially delaying surgical intervention [7]. In this context, intra-articular hyaluronic acid (IA-HA) has gained relevance for its opioid-sparing benefits; by providing a non-addictive alternative for pain management amidst the global opioid crisis, viscosupplementation may reduce the clinical reliance on high-risk analgesics in the OA population [8,9].

Furthermore, indirect costs arising from lost productivity, absenteeism, and premature retirement often surpass direct medical expenses, particularly in the working-age population [3,10,11]. Consequently, the progressive nature of OA not only diminishes the quality of life due to persistent pain and sedentary lifestyle but also exerts immense financial pressure on healthcare systems globally, highlighting the urgent need for effective non-surgical interventions to delay disease progression [1,4,12].

Limitations of conservative management

The primary objective of initial management in knee OA is to alleviate pain and maintain joint function through conservative strategies. Current clinical practice guidelines, such as those from the American Academy of Orthopaedic Surgeons (AAOS), emphasize a multimodal approach including physical therapy, weight loss, and the use of oral analgesics like acetaminophen and nonsteroidal anti-inflammatory drugs (NSAIDs) [1,3]. However, the quality of evidence supporting some conservative modalities varies considerably, which complicates direct comparison of their long-term effectiveness. These interventions often face a “therapeutic ceiling" because, while physical therapy is effective in early stages, its ability to mitigate nonsteroidal symptoms diminishes as joint degeneration progresses and structural damage becomes more severe [3,4].

Furthermore, the long-term pharmacological management of OA presents significant safety challenges. Although effective for acute pain relief, NSAIDs are associated with a well-documented risk of adverse events, including gastrointestinal bleeding, renal impairment, and adverse cardiovascular events [1,13]. These risks are particularly pronounced in the elderly population, who often present with multiple comorbidities and are the primary demographic affected by OA. Consequently, a substantial number of patients remain symptomatic despite conservative efforts, creating a “treatment gap” where they are too symptomatic for oral medications but are not yet candidates for invasive total joint arthroplasty [14,15]. This clinical void, coupled with the inconsistent evidence quality for traditional conservative treatments, underscores the necessity for local, intra-articular therapies that can provide sustained relief with a more favorable safety profile. 

Rationale for viscosupplementation

The therapeutic rationale for viscosupplementation is rooted in the essential physiological role of hyaluronic acid (HA) within the joint environment. In a healthy synovial joint, HA is a high-molecular-weight glycosaminoglycan synthesized by type B synoviocytes and chondrocytes. It serves as a primary constituent of both synovial fluid and the superficial zone of the articular cartilage matrix [4,15]. Within the synovial fluid, HA organizes into a complex network of long, intertwined chains that grant the fluid its unique viscoelastic properties. This allows it to act as a powerful lubricant during slow joint movements, reducing friction to near-zero levels, and as a high-performance shock absorber during rapid loading or impact, effectively protecting the underlying subchondral bone and cartilage [14,16]. Furthermore, HA is theorized to contribute to joint homeostasis by acting as a semi-permeable molecular sieve around the synovium, which regulates the passage of cells and plasma proteins, thereby maintaining a controlled, low-inflammation environment [15,17]. However, it must be acknowledged that estimates of native HA function and many of these homeostatic mechanistic claims, including the sieve effect and viscoinduction, are partly derived from experimental models, which may not fully replicate the complex biomechanics and inflammatory milieu of the human osteoarthritic joint.

In the setting of degenerative joint disease, the concentration and molecular weight of endogenous HA are significantly reduced, leading to increased mechanical friction, accelerated cartilage wear, and a pro-inflammatory state within the synovium [14,18]. Intra-articular (IA) injections of exogenous HA aim to restore these homeostatic properties through a dual mechanism. Initially, the high viscosity of the injected HA provides an immediate “mechanical” benefit by restoring joint lubrication and decreasing friction between the damaged articular surfaces [15,16]. However, the degree to which injected HA replicates the viscoelastic properties of native synovial fluid under real joint-load conditions remains uncertain and is a subject of ongoing investigation. Beyond this, viscosupplementation is believed to trigger a complex “biological” cascade, including the stimulation of endogenous HA synthesis by type B synoviocytes (biosynthesis) and the reduction of cartilage degradation by inhibiting the expression of metalloproteinases and pro-inflammatory cytokines such as IL-1β [13,17,18]. While these pathways are supported by experimental data, they are not uniformly demonstrated across all HA formulations, making it difficult to generalize the magnitude of effect. Statements regarding viscoinduction and chondroprotection in human subjects should therefore be tempered, as their clinical magnitude requires further investigation. Nevertheless, by addressing both the structural and biomechanical drivers of the disease, HA offers a targeted approach that aims not only to alleviate persistent pain but also to potentially delay the overall clinical progression of osteoarthritis [4,12,14].

Ongoing controversies and guideline discrepancies

Despite the widespread clinical use of viscosupplementation, its role in the treatment of knee OA remains one of the most debated topics in orthopaedics and rheumatology. This controversy is primarily fueled by a lack of consensus among major medical societies and the conflicting results of high-profile meta-analyses. While some systematic reviews, including large-scale Cochrane reports, have demonstrated that IA-HA provides statistically significant improvements in pain and function compared to saline placebos [17,18], others have questioned the “clinical significance” of these findings, suggesting the magnitude of effect may not consistently reach the minimum clinically important improvement (MCII) threshold [10,19]. This persistent inconsistency often reflects the substantial variability in study designs and the choice of comparator groups (e.g., diverse saline formulations), which complicates a unified interpretation of the available evidence [20].

The clinical relevance of IA-HA becomes most apparent when addressing the “treatment gap” (the challenging period where patients fail to achieve adequate relief from first-line conservative measures, such as physical therapy and oral analgesics, but are not yet appropriate candidates for surgical interventions like arthroscopy or total knee arthroplasty [13,15]. In this scenario, viscosupplementation functions as a crucial second-line therapeutic bridge. It provides an essential alternative for managing persistent symptoms while offering the potential benefit of delaying the need for more invasive and costly procedures [1,12]. 

These clinical realities are reflected in the stark differences between guidelines. While the American Academy of Orthopaedic Surgeons (AAOS) has historically maintained a cautious or negative stance [3], other influential organizations, such as the European Society for Clinical and Economic Aspects of Osteoporosis and Osteoarthritis (ESCEO), strongly support its use in the OA treatment algorithm [13]. Still, these persistent guideline discrepancies highlight an urgent need for more standardized outcome measures to improve cross-study comparability and help reconcile divergent clinical recommendations [21,22]. This gap between academic skepticism and real-world clinical success underscores the need for a comprehensive review that clarifies the utility of HA through the lens of optimized patient selection and long-term outcomes. 

## Review

Methods

This structured narrative review was conducted following a systematic approach to literature identification to enhance transparency and mitigate the inherent risk of selection bias associated with non-systematic methodologies [23]. A comprehensive search was performed across electronic databases, including PubMed, the Cochrane Library, Google Scholar, and Scopus, following current recommendations for optimal evidence retrieval [24]. The search strategy employed MeSH terms and keywords such as "viscosupplementation," "hyaluronic acid," "knee osteoarthritis," and “clinical outcomes." To ensure a rigorous process, the search and identification phases were guided by the updated Cochrane Handbook for Systematic Reviews [25], covering publications between January 2006 and January 2026.

Selection criteria and reproductibility

To improve reproductibility, specific inclusion and exclusion criteria were applied. Selection criteria focused on high-quality systematic reviews, meta-analyses, randomized controlled trials (RCTs), and clinical practice guidelines. Studies were prioritized if they reported standardized outcomes such as the Visual Analogue Scales (VAS) for pain. Western Ontario and McMaster Universities Osteoarthritis Index (WOMAC) for function. Conversely, we excluded case reports, non-peer-reviewed editorials, and studies with insufficient follow-up (<4 weeks) or non-standardized OA classifications.

Quality assessment and limitations

The evidence was qualitatively assessed by the authors, prioritizing trials with robust blinding protocols and large sample sizes to ensure a balanced representation of the therapeutic landscape. To ensure the scientific rigor of this synthesis, the quality of the review was self-assessed using the SANRA scale (Scale for the Assessment of Narrative Review Articles) [26]. While this approach aims to provide a rigorous synthesis, we acknowledge that the narrative nature of this review inherently lacks the full computational reproducibility of a formal systematic review and may remain susceptible to residual selection bias.

Mechanism of action of viscosupplementation

The therapeutic effect of intra-articular hyaluronic acid (IA-HA) is multifaceted, involving both immediate mechanical improvements and delayed biological responses. In the healthy joint, HA provides the necessary viscosity to lubricate the cartilage surfaces during low-shear movements and elasticity to absorb shocks during high-shear activities [15,16]. In the osteoarthritis joint, where HA is degraded and diluted, viscosupplementation aims to restore intra-articular homeostasis through several pathways: 

Mechanical and Rheological Restoration (Viscoprotection)

The primary and most immediate effect of IA-HA is viscoprotection. As synthesized in a 2025 systematic umbrella review, the restoration of these rheological properties is a prerequisite for stabilizing the intra-articular environment [8]. By reintroducing high-molecular-weight HA, the synovial fluid regains its shock-absorbing and lubricating capacity. This restoration is crucial for decreasing articular wear; the exogenous HA forms a protective coating over the superficial zone of the cartilage, reducing the coefficient of friction and preventing further mechanical abrasion of the internal structure of the extracellular matrix [16].

Furthermore, by restoring the fluid’s elasticity, IA-HA significantly attenuates the transmission of axial loads to the subchondral bone. In a degenerated joint, the loss of viscosity leads to an “impact loading” effect where the subchondral bone absorbs excessive force, triggering bone marrow lesions and subchondral plate thickening [4,15]. Recent Level I evidence from a 2025 Bayesian network meta-analysis confirms that ultra-high (UHMW) and high molecular weight (HMW) formulations demonstrate superior performance in managing this mechanical stress compared to low molecular weight (LMW) products [27]. Viscosupplementation dissipates these forces, thereby reducing the mechanical stress on the highly innervated subchondral bone. This process directly contributes to pain reduction by decreasing the activation of nociceptors (mechanoreceptors) sensitive to intraosseous pressure and mechanical strain [4,15,18].

Biological and Anti-Inflammatory Effects

Beyond simple lubrication, intra-articular hyaluronic acid (IA-HA) acts as a biological modulator of the joint environment. Addressing historical concerns that mechanistic evidence derives primarily from in vitro studies, current translational data from 2025 and 2026 emphasize a high correlation between these molecular pathways and clinical outcomes in advanced OA patients [8,28]. Exogenous HA binds to specific cell surface receptors, most notably CD44 and RHAMM (Receptor for Hyaluronan-Mediated Motility), on synoviocytes and chondrocytes [13,18].

The interaction with CD44 is particularly critical, as it initiates intracellular signaling cascades that inhibit the nuclear factor-kappa B (NF-kB) pathway [29]. This molecular interaction triggers intracellular signaling pathways that lead to several restorative effects. Specifically, IA-HA has been shown to inhibit inflammatory mediators by decreasing the production of pro-inflammatory mediators, such as interleukin-1β (IL-1β) and tumor necrosis factor-alpha (TNF-α), while simultaneously reducing the activity of matrix metalloproteinases (MMPs), specifically MMP-1, 3, and 13, responsible for cartilage breakdown [13,17,28,30].

Chondroprotection and Endogenous Homeostasis 

A pivotal long-term effect of viscosupplementation is viscoinduction: the presence of exogenous HA stimulates Type B synoviocytes to increase the production of native, high-molecular-weight HA. This phenomenon explains why clinical benefits often persist long after the injected substance has been cleared from the joint space [12,14].

This process represents a transition from a catabolic to an anabolic state, as detailed in recent 2026 evidence, which highlights that IA-HA promotes the synthesis of proteoglycans and type II collagen [28]. Additionally, IA-HA exerts a chondroprotective effect by reducing chondrocyte apoptosis, which may stabilize the cartilage matrix and potentially slow the structural progression of osteoarthritis [15,18]. By restarting the joint’s own lubricant factory and creating a molecular shield for chondrocytes, IA-HA helps break the vicious cycle of degradation [8].

Summary of Joint Homeostasis

In summary, these biological pathways suggest that IA-HA functions as more than just a temporary lubricant; it acts as a pivotal modulator of joint health and a protector of articular integrity. By dampening the inflammatory fire (inhibition of mediators), restarting the joint’s own lubricant factory (stimulation of endogenous HA), and creating a protective shield for the remaining cartilage cells (chondroprotection), IA-HA helps to break the vicious cycle of degradation. This shift from a pro-inflammatory to an anabolic and restorative state not only explains the long-lasting symptomatic relief observed in clinical practice but also provides a biological basis for its potential to preserve joint architecture and delay structural decline over time [12,13,18]. Current evidence from 2025 and 2026 high-level syntheses supports this transition, confirming that the clinical efficacy of IA-HA is deeply rooted in its ability to restore the biochemical and mechanical synergy of the joint [8,27,28].

Types of hyaluronic acid used in viscosupplementation

The therapeutic success of viscosupplementation is influenced by a diverse range of formulations. Understanding the chemical and physical properties of these products is essential for optimizing clinical outcomes. 

Molecular Weight and Product Characteristics

Hyaluronic acid (HA) products are categorized by their molecular weight, which significantly dictates their residence time in the joint and their biological signaling. High-molecular-weight (HMW) and cross-linked HA formulations, such as hylan G-F 20, utilize cross-linked HA derived from avian sources to mimic the high viscosity (~6 million Da) of healthy synovial fluid. The main advantage of these products is a prolonged intra-articular half-life and a superior mechanical cushioning effect; furthermore, cross-linking allows for single-injection regimens, which increases patient compliance [14,16].

However, the clinical superiority of these heavy molecules over lighter ones remains a subject of intense academic debate. While a level I Bayesian network meta-analysis (2025) concludes that ultra-high molecular weight (UHMW) and HMW formulations present the highest statistical probability of reducing pain (VAS scale) at six months [27], this theoretical advantage is not uniformly reflected across all trials. Recent comparative studies (2024) have shown that although cross-linked formulas offer the convenience of a single dose, their results in functional scales such as WOMAC can be comparable to those of linear low-weight formulas in short- to medium-term follow-ups, suggesting that clinical response may depend on individual patient factors beyond the product’s molecular weight alone [31]. Despite their efficacy, they are associated with a higher risk of transient local inflammatory reactions, particularly in patients with avian protein sensitivities [32].

Conversely, low and medium molecular weight sodium hyaluronate formulations focus on shorter, linear chains. These products are often praised for their high purity and strong biological interaction with CD44 receptors, potentially restarting the endogenous production of HA more rapidly than heavier molecules [13,15]. Their primary drawback is a shorter half-life within the joint, typically requiring multiple weekly injections to achieve a cumulative therapeutic effect.

Origin: Avian vs. Biofermentative Sources

The source of HA remains a point of clinical relevance regarding safety and purity. Avian-derived HA formulations such as hylan G-F 20 (Synvisc, Synvisc-One) are refined from rooster combs and have a long clinical track record demonstrating consistent results in pain reduction and functional improvement. While highly effective due to their high molecular weight, there is a theoretical risk of hypersensitivity in patients allergic to avian protein, necessitating careful patient screening [13,17].

In contrast, modern products produced via bacterial fermentation, such as those derived from Streptococcus equi, like the Ostenil range or certain high-purity sodium hyaluronates like Suprahyal (depending on the regional formulation), offer a “non-animal” alternative. These formulations reduce the risk of immunogenic reactions and ensure high batches of purity, which has made them the current standard in many international markets [15,32]. Despite the clinical preference for biofermentative products due to their controlled purity, it is fundamental to acknowledge that the scientific evidence lacks robust “head-to-head” safety studies directly comparing both origins. Most current literature is based on post-marketing pharmacovigilance reviews, and while immunogenic reactions are theoretically lower in bacterial sources, the lack of direct comparative clinical trials prevents establishing an absolute and definitive safety superiority in the evidence-based practice environment [8].

Specialized and Combined Formulations

The evolution of HA has led to specialized presentations for different clinical needs, including variations in concentration. One of the most notable innovations in 2025-2026 is the development of stable hybrid cooperative complexes (HCC), which integrate high and low molecular weight chains through a patented thermal treatment without the need for chemical cross-linking agents. This technology allows for exceptionally high HA concentrations while maintaining manageable viscosity, optimizing both biological integration and mechanical cartilage protection [8]. While 1.0% sodium hyaluronate solutions are standard for volume-based lubrication (Ostenil, 2.0 mL; Ostenil Mini, 1.0 mL), higher concentrations like 2.0% sodium hyaluronate (Ostenil Plus, 2.0 mL; Ostenil Tendon, 2.0 mL) provide a more dense “scaffold” effect, which is particularly beneficial in smaller joints or for managing the complex mechanical stress of peritendinous tissues [15]. The transition toward single-dose, high-concentration, and cross-linked products represents a significant progress for the patient, as it reduces the number of clinical visits and the cumulative risk of infection associated with multiple needle penetrations [16,18].

Finally, the current frontier of viscosupplementation is shifting toward combination therapies. Literature from 2026 highlights the use of HA in combination with biological agents such as platelet-rich plasma (PRP) or extended-release corticosteroids. This multimodal approach seeks not only to lubricate the articular compartment but also to synergistically modulate the inflammatory environment, providing faster pain relief and potentially more durable chondroprotection than HA administered as monotherapy [28]. The characteristics and clinical differences between these various hyaluronic acid formulations are summarized in Table 1.

**Table 1:** Comparison of intra-articular hyaluronic acid formulations based on molecular weight, production source, and clinical considerations. HA: Hyaluronic Acid; HMW: High-Molecular-Weight; LMW-MMW: Low/Medium-Molecular-Weight; OA: Osteoarthritis; CD44: Cluster of Differentiation 44 receptor. Note: Comparative analysis of common hyaluronic acid formulations. Differences in molecular weight, origin, and administration regimens contribute to the clinical variability observed in treatment outcomes. Source data: [6-10,13].

Feature	High-Molecular-Weight (HMW) / Cross-linked	Low/Medium Molecular Weight (LMW-MMW) / Linear
Origin / Source	Avian-derived (Refined from rooster combs) +2	Biofermentation (Streptococcus equi)
Common Brands	Synvisc, Synvisc-One, Hylan G-F 20 +2	Suprahyal, Ostenil, Hyruan Plus +1
Primary Mechanism	Mechanical: Superior viscoprotection and shock absorption +1	Biological: Higher affinity for CD44 receptors; stimulates endogenous HA +1
Dosing Regimen	Single intra-articular injection +4	Multiple weekly injections (typically 3 to 5) +1
Clinical Advantage	Longer joint residence time; higher patient compliance	High purity; reduced risk of immunogenic/avian reactions +1
Primary Risk	Higher incidence of local "flare-ups" or pseudosepsis +3	Cumulative risk of infection due to multiple needle entries +1

Clinical evidence and outcomes

The clinical efficacy of intra-articular hyaluronic acid (IA-HA) has been extensively documented, demonstrating a distinct therapeutic trajectory that differentiates it from other intra-articular treatments like corticosteroids. Meta-analyses of randomized controlled trials indicate that while corticosteroids offer a rapid but short-lived onset of action, the pain-relieving effect of HA follows a more gradual and sustained curve. This “therapeutic window” begins around week four, reaches its maximum peak between weeks 8 and 12 post-injection, and maintains a clinically significant benefit for up to 24 to 26 weeks [4,17]. A recent systematic umbrella review from 2025 confirmed this duration, highlighting that IA-HA significantly outperforms placebo in both pain reduction and functional improvement at the 26-week mark, a phenomenon increasingly attributed to the restoration of synovial homeostasis [8].

The long-term evidence provided by the AMELIA project (Osteoarthritis Molecular Evaluation of Likely Impact of Alginate/Hyaluronan) is perhaps the most compelling argument for the use of IA-HA in chronic management. In this 40-month study involving repeated cycles of HA, researchers observed that the clinical benefit was not only sustained but actually improved with each subsequent cycle. The “responder rate” increased progressively over time, suggesting that repeated applications may interfere with the underlying pathophysiology of osteoarthritis, providing a longitudinal symptom control that surpasses the effect of a single isolated cycle [12]. This longitudinal benefit is further supported by 2026 real-world evidence from post-market clinical follow-up (PMCF) studies, which demonstrate sustained efficacy and safety of high-purity sodium hyaluronate up to 28 weeks, extending the previously accepted therapeutic window [33].

Evidence by Formulation and Dosing Regimen

When transitioning from general outcomes to specific formulations, the evidence highlights important nuances regarding efficacy and administration. Data from large-scale reviews of US-approved products (e.g., Hylan G-F 20) consistently show a robust effect size. High-molecular-weight formulations tend to provide a more durable mechanical "cushion," which translates into significant improvements in the Western Ontario and McMaster Universities Osteoarthritis Index (WOMAC) pain and function scores [15]. A 2025 Level I Bayesian network meta-analysis reinforces this, concluding that Ultra-High Molecular Weight (UHMW) and High Molecular Weight (HMW) formulations have the highest statistical probability of being the most effective treatments for pain reduction (VAS) at six months [27].

Specifically, single-injection cross-linked products have demonstrated non-inferiority when compared to traditional three-week or five-week cycles, offering a high degree of efficacy with the added benefit of reducing the risk of iatrogenic infection and improving patient convenience [14,16]. Comparative studies in 2024 and 2025 confirm that cross-linked HA provides comparable clinical outcomes to linear formulations in terms of VAS and WOMAC scores, with some single-dose regimens showing a significant reduction in pain scores (averaging a decrease of over 4 points on the VAS scale) by the six-month follow-up [31,34].

In contrast, linear and medium-molecular-weight formulations remain the gold standard for many clinicians due to their well-characterized biological interaction with the synovial receptors. While these products often require multiple injections, typically administered in three to five doses, they show a consistent ability to reduce joint stiffness and improve mobility in patients with moderate osteoarthritis, specifically those classified as Kellgren-Lawrence grades II and III [13,18].

Across all formulations, the safety profile remains excellent. Although HMW cross-linked products have been associated with a slightly higher incidence of “flare-ups” or transient local swelling (pseudosepsis) when compared to linear low-molecular-weight HA, these events are typically self-limiting and do not compromise the long-term success of the treatment [15,32]. Modern high-purity and cross-linked formulations continue to demonstrate a superior safety profile in 2025-2026 surveillance data, with a negligible incidence of serious treatment-related adverse events, confirming their suitability for long-term management of chronic knee osteoarthritis [33,34].

Advanced and combination therapies

The evolution of viscosupplementation has transitioned from a simple monotherapy to complex multimodal strategies designed to address the biochemical and mechanical environment of the osteoarthritic joint simultaneously. Current evidence from 2025 and 2026 highlights that combining intra-articular hyaluronic acid (IA-HA) with other biological and pharmacological agents can provide synergistic effects that surpass the efficacy of HA alone [28].

HA and Corticosteroids

The combination of IA-HA and corticosteroids (CS) is one of the most widely used strategies to optimize the therapeutic window. While CS provides rapid, high-potency anti-inflammatory relief within the first week, IA-HA ensures a sustained symptomatic benefit from week 4 through week 26. Recent high-level evidence suggests that this combination effectively "quenches" the acute inflammatory fire while providing the necessary rheological support to the joint, leading to superior WOMAC scores in the medium term compared to either agent in monotherapy [8,28].

HA and Platelet-Rich Plasma (PRP)

The biological synergy between IA-HA and platelet-rich plasma (PRP) represents a significant advancement in regenerative medicine. HA serves as a bio-scaffold that stabilizes the joint environment, while PRP provides a high concentration of growth factors (such as TGF-β and PDGF) that stimulate chondrocyte proliferation and extracellular matrix synthesis. Systematic reviews from 2026 indicate that the IA-HA + PRP combination significantly enhances the anti-inflammatory environment by inhibiting the NF-κB pathway more effectively than HA alone, resulting in better functional outcomes and potential structural preservation at 12 months [28].

Clinical Considerations and Potential Drawbacks

While combination therapies offer significant synergistic potential, they are not without clinical "caveats" that must be carefully managed. A critical concern with intra-articular corticosteroids (CS) is the risk of iatrogenic crystal-induced synovitis. Certain CS formulations, particularly those with low solubility intended for long-term effect (such as triamcinolone hexacetonide), can precipitate and form microcrystals within the synovial space. As noted in recent 2025 safety reviews, these crystals can trigger an acute inflammatory "flare-up" that mimics septic arthritis, leading to significant post-injection discomfort and potentially accelerating chondrocyte damage if repeated frequently [8].

Furthermore, evidence from 2026 emphasizes the "chondrotoxicity" risk associated with prolonged corticosteroid use. While HA provides a protective scaffold, the metabolic interference of CS with chondrocyte activity can lead to a net loss of cartilage volume over time if the dosing interval is too short. Similarly, for HA and PRP combinations, the primary "cons" involve the lack of standardization in PRP preparation (leukocyte-rich vs. leukocyte-poor) and the higher cost-benefit ratio, which may limit its accessibility in diverse clinical settings [28]. Therefore, the transition to combination therapy should be individualized, balancing the rapid relief of corticosteroids against their potential for crystalline-induced flares and structural interference.

New Technologies: Hybrid Cooperative Complexes (HCC)

Innovation in HA molecular engineering has led to the development of stable hybrid cooperative complexes (HCC). Unlike traditional cross-linking, which relies on chemical agents, HCCs utilize a patented thermal process to combine high-molecular-weight and low-molecular-weight HA chains. This results in a high-purity formulation with high concentration and low viscosity, optimizing both biological signaling through CD44 receptors and mechanical viscorestoration. Evidence from 2025 suggests that HCCs provide a more robust "viscoinduction" effect, promoting the joint's endogenous HA production more efficiently than standard linear formulations [8].

Emerging Agents and Future Biologics

Beyond traditional combinations, current research is exploring the integration of HA with agents such as polydeoxyribonucleotides (PDRN), mesenchymal stem cells (MSCs), and even botulinum toxin A for patients with centralized pain components. These "advanced viscosupplements" aim to provide a comprehensive treatment that addresses pain, inflammation, and structural decline through diverse molecular pathways, marking the future of personalized osteoarthritis management [28].

Indications and patient selection

The clinical success of viscosupplementations is highly dependent on precise patient selection, as the therapeutic response varies significantly according to the stage of the disease and the patient’s long-term goals. Evidence strongly suggests that the “therapeutic sweet spot” for intra-articular hyaluronic acid (IA-HA) lies in patients with mild-to-moderate knee osteoarthritis, specifically those classified as Kellgren-Lawrence (KL) grades II and III [13,18]. However, predicting an individual patient’s response remains a significant clinical challenge due to the inherent heterogeneity in symptom severity and the varying rates of structural degeneration. Recent 2025-2026 evidence emphasizes that while radiographic grading is a fundamental framework, it does not guarantee a uniform therapeutic outcome, as pain perception and functional decline often decouple from imaging findings [8,33].

The “Therapeutic Sweet Spot”: KL Grades II and III

In KL Grade II, which is characterized by early osteophyte formation and possible joint space narrowing, IA-HA acts as a biomodulator that can effectively dampen the initial inflammatory cascade and preserve the relatively healthy cartilage matrix remaining [15,18]. Despite this, the “clinical reality” is that even within KL II populations, the responder rate is influenced by the patient’s baseline inflammatory state and metabolic profile, necessitating a personalized clinical approach rather than a one-size-fits-all protocol [33].

For patients with KL Grade III, defined by moderate multiple osteophytes, definite joint space narrowing, and some sclerosis, the mechanical and rheological properties of HA are vital to compensate for the significant loss of endogenous viscoelasticity, providing symptomatic relief that often exceeds the results of oral analgesics [4,13]. Current level I Bayesian network meta-analyses reinforce that while these patients benefit most from high-molecular-weight formulations, individual success is highly dependent on the degree of subchondral bone involvement and the presence of bone marrow lesions, which may limit the efficacy of standard viscosupplementation [27].

Delaying Surgical Intervention and the “Treatment Gap”

Furthermore, a critical indication for viscosupplementation is the management of patients whose primary objective is delaying surgical intervention. Total knee arthroplasty (TKA), while effective, is an invasive procedure with a finite lifespan for the prosthesis, making it less ideal for younger, active patients or for those with high perioperative risks due to comorbidities [1,12]. 

In this context, IA-HA functions as a “bridge therapy.” Long-term data, such as the one obtained from the AMELIA project, demonstrate that repeated cycles of HA can significantly extend the time a patient remains functional and pain-free before requiring a joint replacement [12]. This is particularly relevant for patients in the treatment gap” (those who have exhausted conservative options like physical therapy and NSAIDs but wish to postpone surgery for various reasons) [14,15]. Real-world performance data from 2026 confirm that high-purity HA formulations can sustain clinical performance for up to 28 weeks, effectively narrowing this treatment gap and providing a viable non-surgical alternative for a broader range of patients [33].

Surgical Preservation and Complementary Use

Additionally, viscosupplementation can be integrated into a broader surgical preservation strategy. For some patients, IA-HA serves as a precursor or a complementary treatment to other less invasive surgical interventions, such as arthroscopic joint debridement or meniscus repair [15,18]. By improving the intra-articular environment and reducing synovial inflammation, HA injections can optimize joint conditions before or after these procedures, further extending the functional life of the native joint and delaying the progression toward more radical and invasive reconstructive surgeries [4,12,13]. This multimodal strategy, increasingly supported by 2026 perspectives on combination therapies, highlights the role of HA not just as a lubricant but as a foundational component of a personalized joint preservation plan [28].

Safety profile and complications

Viscosupplementation is widely recognized for its excellent safety profile, especially when compared to the systemic risks associated with chronic oral medication. A 2025 systematic umbrella review reinforces that intra-articular hyaluronic acid (IA-HA) effectively avoids the gastrointestinal, renal, and cardiovascular complications commonly associated with long-term use of non-steroidal anti-inflammatory drugs (NSAIDs) [8]. Furthermore, evidence from 2020 highlights a significant public health benefit: IA-HA therapy is associated with a substantial decrease in the cumulative burden of opioids and intra-articular corticosteroids, thereby mitigating the systemic and chondrotoxic risks inherent to these pharmacological alternatives [9].

Systematic reviews and meta-analyses, including the report made by Honvo et al. [32], indicate that there is no significant increase in the risk of serious adverse events (SAEs) following IA-HA injections compared to saline placebos [15,32]. This high safety threshold is further validated by recent 2026 post-market clinical follow-up (PMCF) studies, which reported zero serious treatment-related adverse events over an extended 28-week surveillance period, confirming the long-term safety of high-purity sodium hyaluronate in diverse patient populations [33].

The most common complications are transient local reactions, such as mild pain at the injection site, localized swelling, or joint stiffness (effusion). These “flare-ups" are typically self-limiting and resolve within 24 to 72 hours without long-term sequelae [32]. Current safety evaluations from 2025 specifically regarding cross-linked HA formulations indicate that these local reactions remain infrequent and mild, with no statistical difference in the safety profile compared to linear formulations, even when using single-dose high-concentration products [34].

A rare but more intense reaction, often referred to as pseudosepsis (a non-infectious inflammatory reaction), has been more frequently associated with high-molecular-weight cross-linked products, though it remains an uncommon clinical occurrence [17,32]. However, modern high-purity and biofermentative manufacturing processes have significantly mitigated the risk of immunogenic reactions compared to early-generation avian-derived products [33]. To minimize risks, clinicians must ensure strict aseptic techniques during the procedure and rule out pre-existing joint infections or cutaneous lesions at the injection site. 

Current guidelines and controversies

The clinical utility of viscosupplementation remains one of the most polarized topics in musculoskeletal medicine. This controversy is driven by the discrepancy between “statistically significant” results and “clinically important” improvements. Meta-analyses by Rutjes et al. [10] and Pereira et al. [1] have argued that while IA-HA outperforms saline placebos, the effect size often falls below the minimum clinically important improvement (MCII) threshold, leading to a cautious or “strong recommendation against” its use in the American Academy of Orthopaedic Surgeons (AAOS) guidelines [3,19].

However, the scientific landscape in 2025 and 2026 has provided a more nuanced perspective. High-level evidence from Bayesian network meta-analyses now demonstrates that when HA is differentiated by molecular weight, High (HMW) and Ultra-High Molecular Weight (UHMW) formulations show a significantly higher statistical probability of achieving clinically relevant pain reduction compared to lower-weight variants, challenging the “class effect” assumptions used in earlier negative guidelines [27].

This “guideline gap” is further exacerbated by methodological challenges in clinical trials. Critics of the negative guidelines argue that the intra-articular saline injection used as a placebo is not a true inert control, because the volume of saline itself can dilute inflammatory cytokines and provide a transient “washout” effect, which artificially narrows the therapeutic margin between the control group and the HA group [15]. Recent systematic umbrella reviews emphasize that this "saline effect" may underestimate the true therapeutic gain of HA by up to 30%, a factor that must be corrected in future evidence syntheses to accurately reflect its clinical value [8].

While AAOS focuses on a rigid interpretation of statistical thresholds, organizations such as the European Society of Clinical and Economic Aspects of Osteoporosis and Osteoarthritis (ESCEO) and the International Osteoarthritis Research Society (OARSI) advocate for a more pragmatic, patient-centered approach, recognizing that for patients who cannot tolerate NSAIDs or for whom physical therapy has plateaued, even a modest reduction in pain can be the difference between maintaining mobility and progressing to a sedentary state [13,18]. This pragmatic view is increasingly supported by the significant economic burden associated with moderate to severe knee osteoarthritis. Evidence from 2024 indicates that the direct and indirect costs of managing advanced OA, including healthcare resource use and lost productivity, are substantial, making any intervention that preserves function and delays surgery a critical component of a cost-effective healthcare strategy [11].

This divergence in medical literature ultimately highlights the role of viscosupplementation as a critical intermediate clinical milestone. Rather than being viewed as a “final cure” or a failed intervention, IA-HA is positioned as the definitive non-surgical bridge that fills the void between basic conservative management and irreversible surgical pathways. In the clinical algorithm, it serves as a “safety valve” that allows both the patient and the surgeon to exhaust all joint-preserving options before committing to a total joint replacement [1,12].

A pivotal clinical advantage emerging in 2020-2026 data is the role of IA-HA in the "opioid-sparing" strategy. IA-HA therapy has been shown to significantly decrease the subsequent burden of opioids and intra-articular corticosteroids, providing a safer alternative in the longitudinal management of chronic pain and reducing the risks associated with these more aggressive pharmacological interventions [9].

By acting as this final line of defense in joint preservation, viscosupplementation provides a structured transition that optimizes the timing of surgery (ensuring that invasive procedures are reserved for the most refractory cases while maintaining the patient’s quality of life during the intervening years [4,13,15]. This “middle-ground” positioning not only justifies its continued use in modern orthopedics but also underscores the necessity of personalized treatment pathways that prioritize the longevity of the native joint. As the field evolves toward regenerative approaches, including the development of advanced HA-based hydrogels for cartilage tissue engineering, IA-HA remains the foundational pillar for both current symptomatic relief and future biological restoration [35].

## Conclusions

Viscosupplementation with hyaluronic acid represents a safe, effective, and minimally invasive therapeutic option for patients suffering from mild-to-moderate knee osteoarthritis (Kellgren-Lawrence grades II-III). By restoring the rheological properties of the synovial fluid and modulating the intra-articular inflammatory environment, IA-HA provides sustained pain relief and functional improvement. While a six-month therapeutic window is a commonly observed clinical milestone, it is essential to recognize that the duration of benefit is not uniform; individual outcomes are highly variable and depend on a complex interplay of patient-specific factors and baseline disease activity.

While academic controversies persist due to variations in meta-analysis methodologies and guideline criteria, long-term clinical evidence suggests that repeated treatment cycles offer cumulative benefits and can significantly delay the need for total joint replacement. However, the observed variability between individual clinical trials necessitates a cautious interpretation when extrapolating these results to broad, heterogeneous clinical populations. Practitioners should adopt a conservative approach, acknowledging that study-specific limitations and patient diversity may influence the generalizability of reported success rates. Ultimately, the success of viscosupplementation depends on appropriate patient selection (targeting those in the “treatment gap” who have failed initial conservative measures) and the use of high-quality formulations. Current evidence confirms that high-purity and cross-linked formulations can sustain clinical performance for up to 28 weeks, providing a critical "safety valve" that reduces the systemic burden of opioids and corticosteroids while mitigating the substantial economic impact of advanced osteoarthritis. As the field evolves toward regenerative hydrogel technologies and tissue engineering, IA-HA remains a vital, evidence-based pillar in the personalized preservation of joint function and the enhancement of patient quality of life.
